# Supplementary material for: Patterns of Intron Gain and Loss in Fungi
Source: PLoS Biol. 2004 Nov 30;2(12):e422. doi: 10.1371/journal.pbio.0020422 (PMC532390; doi:10.1371/journal.pbio.0020422)
Supplement: Table S1 — Also available at http://genes.mit.edu/NielsenEtAl/. (4.3 MB ZIP). [file pbio.0020422.st001.zip › NielsenEtAl/html/942.html]

AN3626.1.NCU03194.1.MG01256.1.FG10669.1


```
 CLUSTAL W (1.82) Multiple Sequence Alignments - Introns Inserted


Sequence 1: NCU03194.1	624 aa
Sequence 2: MG01256.1	671 aa
Sequence 3: FG10669.1	595 aa
Sequence 4: AN3626.1	572 aa
Alignment Length: 702 aa
Number Identitical Residues: 219 aa
Alignment Score (without introns) 11590


MG01256.1 	---~-----MSQETVVGLLGGGQLGRMLCEAAGPLGFPIAILDEGNSPAKQALHG-PHVD
NCU03194.1	---~----MGSSNPVIGLLGGGQLGRMLCEAANPLGIDIAILDEKNSPAKQAHNTNRHVT
FG10669.1 	---~-----MSGKPELGLLGGGQLGRMLCEAASPLEVEIAILDAQDAPAKQVSRSKYHVD
AN3626.1  	MNV2PPLLRMWNSRKVGVLGGGQLGRMFVESANRLNIQCNILDAENSPAKQISAHDDHVT
          	 .  ..      .  :*:*********: *:*. * .   ***  ::****    . ** 

MG01256.1 	GSFKDPQKIRELARKCDVLTVEIEHVDTEVLEEIATKGVEVQGS-----MKKIPVHPSWE
NCU03194.1	GSFKDPVKIRELAARSDYLSVEIEHVETEVLEDIEKNGVEIKLADGSVAIHKPPIHPSWR
FG10669.1 	GSFKDPAKIRELASHCKVLSVETEHIETAVLEELSKEG-------------KVVVYPSWK
AN3626.1  	GSFKEREAVRELAKKCDVITAEIEHVDTYALEEIAS---------------QVQIEPSWQ
          	****:   :**** :.. ::.* **::* .**:: .               :  : ***.

MG01256.1 	TIRLIQDKFLQKEHYK--KHKLPIAEQVAIESG--DAMLSSLKDAGRKFGYPLMLK0ARK
NCU03194.1	TIRLIQDKYLQKEHFRTSKTKIPIADQMAIESG--ESALKSLQEAADKFGFPFMLK~ARK
FG10669.1 	TLRLIQDKYEQKDYLG--KQGIPIAEQVAVQASGSDDMRAALKDISNKYGLPFMLK~SRK
AN3626.1  	AIRTIQNKFNQKEHLA--KHGIPMAEHRELVEN----TPEQLAKAGEELGYPLMLK~SKT
          	::* **:*: **::    *  :*:*::  :  .        * . . : * *:*** ::.

MG01256.1 	GSYDGRG~NFKISSEADLEHAVKELGSLSLYAEKWCKF~VQELAVMVIRTEDDQGNLKSL
NCU03194.1	GSYDGRG~NFKVNSAEDFAPAIEALGKLPLYAEKWAPF~VKELAVMVIRTEDDEGNLRKC
FG10669.1 	DSYDGRG~NIKIANDNDIETAVTEFGNLQCYAEKYVPF~QRELSVIVIRTEDADGKTKRL
AN3626.1  	MAYDGRG1NYRVNSKDDIPDALEFL--------KGRPL2SQELAVMVIKTKD------AV
          	 :***** * :: .  *:  *:  :        *   :  :**:*:**:*:*        

MG01256.1 	AAYPAVETIHEDSICTKTFMPPRNVSDEICEKARETARQVISTISGRGVFAVEMFLLEDG
NCU03194.1	VAYPAVETIHEDSICTKVFMPPRGVDDNVCEQARKLACDVISTLWGRGVFAVEMFLLEDG
FG10669.1 	VPYPAVETVHEDNVCSRVYMPPRDTPDAVSKRAQEVAVSVVEKLWGRGVFAVEMFVTENN
AN3626.1  	LSYPTVETVQEDSICKLVYAPARNVPEHINQAAQKLARKAVATFEGKGAFGVEMFLMNDN
          	 .**:***::**.:*. .: *.*.. : : : *:: * ..: .: *:*.*.****: ::.

MG01256.1 	~SILVNEVAPRPHNSGHYTIEAVPYF~SQYKAQLYALLDLPIPKQLKPRVASAIMINILG
NCU03194.1	1SLMINEVAPRPHNSGHYTIEAVPYM~SQYKAQLYSVLDLPVPDELTPRVSSSIMLNILG
FG10669.1 	~DILVNEIAPRPHNSGHLTIEAVPYM2SQYKAQLTSILDEPLPETLEPHVSSSIMINILG
AN3626.1  	~SLLLCELASRVHNSGHYTIEGT-AL~SQFDAHLRAILDLPIPPESLQLRQPSIMLNIIG
          	 .::: *:*.* ***** ***..  : **:.*:* ::** *:*        .:**:**:*

MG01256.1 	GAGPESHNDLVETAARSFEDDIDVHVHLYGK-ASKPGRKIGHITITSEL-RIAELQKKSE
NCU03194.1	GAAPNSHHKLARLARTTYGDDFDVYLHLYNK-ESKPTRKIGHITLTGFS-SIEDLEKAAE
FG10669.1 	GASPESHIPLVNKAKSMFAPKIGVYPHLYGK-QSKPGRKIGHITLTGLEGTIKDLEKFAQ
AN3626.1  	GSAPDTHLKAAEAALSIPN----ASIHLYSKGDAKPGRKMGHVTVTAST--MHKAEEYIQ
          	*:.*::*   .. *         .  ***.*. :** **:**:*:*.    : . ::  :

MG01256.1 	HLFVLADAMRAERLKAASAQLRPQESVVKSSSSAP---------SKTDALVLVTMGSDSD
NCU03194.1	PFISMVDQIREERLAASAEQLRPQEEAKASNKTNPGGANPVEGNNSDQPLVLVTMGSDSD
FG10669.1 	PLVQLAADMRQERIEAKSKAMRPEQAVVKT---------------AKDPLVLVTMGSDSD
AN3626.1  	PLIDVVDEIRSQRQDIKTN---PAPSGVTK----------------PPPQVAVMMGSDSD
          	 :. :.  :* :*    :    *      .                  . * * ******

MG01256.1 	LPVMKAGLDILK-QFGVPYEVRITSAHRTPNLMGDVAAEAAGRGIKVAIAA0AGGAAHLP
NCU03194.1	LPVLKAGLDILK-EFKVPYALDITSAHRTPKYMMKVAGEAAKKGIKVIIAA~AGGAAHLP
FG10669.1 	LPVLKAGLDILT-QFGVPWEVDITSAHRTPVKMGDVAVAAADRGIKVIIAA~AGGAAHLP
AN3626.1  	LKTLVPGLKLLRDKFGIEPAVDITSAHRTPTFMAEYSASAAARGIKVIIAA~AGGAAHLP
          	* .: .**.:* .:* :   : ********  * . :  ** :**** *** ********

MG01256.1 	GMFASHCPIPVIGVPVKATHLDGEDSLLSIVQMP~RG~VPVATVGINNSTNAALLAIRML
NCU03194.1	GMISSETPLPVIGVPVKATHLDGLDSLLSIVQMP0RG~VPTATVGINNSTNAALLAIRIL
FG10669.1 	GMISAYTPLPVIGVPVKATHLDGMDSLLSIVQMP0RG~VPTATVGINNSTNAALLAIRIL
AN3626.1  	GMAAAHTALPVIGVPVKGSSLDGVDSLYSIVQMP~RG1VPVATVGINNSINAALLAARII
          	** ::  .:********.: *** *** ****** ** **.******** ****** *::

MG01256.1 	GSFIPKYQDQMKSYQQEME-DAVLAKDEELRGIGYESYLAKMA1SPNRANTGQAGIPKRT
NCU03194.1	GSFIPQYQEHMSRYQLQME-EAVIEKGGKLRTGGDVAYLEGMK~KTK-------------
FG10669.1 	GAFIPEYLEKMKGYQTDIG-EQVNGKATRLRESDVESYLAQMK~KG--------------
AN3626.1  	GAFDPDVQRKVEEYAEDARKENMDMKNVKMQDLGWEKYFNQM-~----------------
          	*:* *.   ::. *  :  .: :  *  .::  .   *:  *                  

MG01256.1 	MAPCGSDKVSIMRGNGATRG1KGQKRRQKQKKYQQPGLTIQIAGWWCIL0AIAH
NCU03194.1	--------------------~----------------------------~----
FG10669.1 	--------------------~----------------------------~----
AN3626.1  	--------------------~----------------------------~----
          	
```
